# Cranial autonomic symptoms: prevalence, phenotype and laterality in migraine and two potentially new symptoms

**DOI:** 10.1186/s10194-022-01389-w

**Published:** 2022-01-29

**Authors:** Nazia Karsan, Karthik Nagaraj, Peter J. Goadsby

**Affiliations:** 1 Headache Group, Division of Neuroscience, Wolfson Centre for Age Related Diseases, Institute of Psychiatry, Psychology and Neuroscience, King’s College London, London, UK; 2 South London and Maudsley Biomedical Research Centre, King’s College Hospital, London, UK; 3 Department of Neurology, Bangalore Medical College and Research Institute, Bangalore, India; 4 Department of Neurology, University of California, Los Angeles, Los Angeles, CA USA

**Keywords:** Migraine, TAC, Cranial autonomic, Voice change, Throat swelling, Headache, Trigeminal

## Abstract

**Background:**

Whilst cranial autonomic symptoms (CAS) are typically associated with trigeminal autonomic cephalalgias (TAC’s), they have also been reported in migraine. Identification and understanding of these symptoms in migraine is important to ensure timely diagnosis and effective management.

**Methods:**

Migraineurs seen in a tertiary headache service between 2014 and 2018 (*n* = 340): cohort one, and a separate cohort of headache patients seen between 2014-May 2021 reporting voice change, or throat swelling, or both, as CAS were selected (*n* = 64): cohort two. We performed a service evaluation of our records regarding age, sex, diagnosis, headache and CAS frequency and laterality as acquired from the first consultation, during which a detailed headache history is taken by a headache trained physician.

**Results:**

Cohort 1: Mean age 43 (range 14–94, SD 15). The most common diagnosis was chronic migraine (78%). Median monthly headache frequency was 26 days (IQR 15–75). At least one CAS was reported in 74%, with a median of two (IQR 0–3). The most common were nasal congestion (32%), lacrimation (31%) and aural fullness (25%). Most patients reported their most common headache as unilateral (80%) and with it strictly unilateral CAS (64%). There was a positive association between headache and CAS laterality (*χ*^2^_1_ = 20.7, *P* < 0.001), with a positive correlation between baseline headache frequency and number of CAS reported (*r* = 0.11, *P* = 0.047). Cohort two: mean age 49 (range 23–83, SD 14). Diagnoses were chronic migraine (50%), chronic cluster headache (11%), undifferentiated continuous lateralised headache (9%), SUNCT/SUNA (8%), hemicrania continua (8%), episodic migraine (8%), episodic cluster headache (3%) and trigeminal neuropathies (3%). Most (89%) described trigeminal distribution pain; 25% involving all three divisions. Throat swelling was reported by 54, voice change by 17, and both by 7. The most common CAS reported were lacrimation (*n* = 47), facial swelling (*n* = 45) and rhinorrhoea (*n* = 37). There was significant agreement between the co-reporting of throat swelling (*χ*^2^_1_ = 7.59, *P* = 0.013) and voice change (*χ*^2^_1_ = 6.49, *P* = 0.02) with aural fullness.

**Conclusions:**

CAS are common in migraine, are associated with increasing headache frequency and tend to lateralise with headache. Voice change and throat swelling should be recognized as possible parasympathetically-mediated CAS. They may be co-associated and associated with aural fullness, suggesting a broadly somatotopic endophenotype.

## Background

Cranial autonomic symptoms (CAS) have been typically associated with the trigeminal autonomic cephalalgias (TAC’s), in which they form part of the diagnostic criteria, and are usually lateralised to the side of the pain [1]. However, it has been increasingly recognised that these symptoms can also occur in migraine and should not deter from the diagnosis [2–13]. The laterality of these symptoms in migraine has also been examined, and co-lateralisation with pain akin to the TAC’s has also been demonstrated in some studies, although clearly less commonly than in the TAC’s [4, 6, 11]. This lateralisation may be predictive of triptan treatment response [14–16]. Similarly, photophobia and phonophobia are also less commonly lateralised in migraine compared to the TAC’s [17].

In an experimental study, we have previously demonstrated that CAS can precede headache in migraine [18], and indeed can occur or persist following headache resolution [19]. Others have shown similar findings in cluster headache, where CAS can occur early before pain onset [20–22], and along with other symptoms may be predictive of an impending bout [23]. Clearly, identification and recognition of these symptoms as part of the broader migraine phenotype is important to allow timely diagnosis and effective headache management, in particular in not infrequent, sometimes challenging diagnostic situations for headache physicians, when patients with cluster headache or another TAC have co-existent migraine, and in those with migraine with short attacks.

In primary headache disorders, pain from the structures of the head and neck is thought to be as a result of trigeminovascular activation; that is the system of nociceptive bipolar nerve fibres originating in the trigeminal ganglion (TG), with the peripheral innervation of dural vessels and large cranial vessels and venous sinuses (causing vascular dilatation), and a central projection to the caudal brainstem or high cervical cord [24, 25]. Cervical dorsal root ganglia also innervate the dura [26, 27]. The central pathway runs through the trigeminocervical complex (TCC), and the neuronal inputs from the peripheral and central afferent pathways converge here, with subsequent projection to higher brain areas including the thalamus and cerebral cortex [28]. Activation of the TCC leads to neuronal activation within the superior salivatory nucleus (SSN) in the pons [29, 30]. An afferent arc in the trigeminal nerve (mainly V1), a reflex parasympathetic connection to the SSN in the pons and an efferent arc in the facial nerve, via the sphenopalatine ganglion (SPG) and greater superficial petrosal nerve, are thought to mediate CAS observed in the TAC’s and migraine, using vasoactive intestinal peptide (VIP) [31] and other neurotransmitters [32]. CAS can be provoked by V1 pain stimulation in healthy volunteers also [33].

Parasympathetically-mediated CAS include lacrimation, conjunctival injection and rhinorrhoea, and sympathetic impairment with ptosis, miosis, sweating and flushing are also well reported. Descending control over the TCC and SSN occurs via the periaqueductal grey (PAG) and hypothalamus, amongst other diencephalic areas, such as the locus coeruleus (LC) [34], and may be a plausible mechanism for top-down activation of the SSN without TCC activation as an explanation for why CAS can present without pain [34].

We were interested in looking into the prevalence of CAS in our clinical cohort of migraineurs, as we suspect that symptoms are more common that often reported in the literature, as systematic questioning may be less clinically pressing when the migraine diagnosis is more obvious. In our clinical history taking, the questions are standardised regardless of likely underlying diagnosis so we felt that this may increase patient reporting of symptoms, despite it possibly leading to a degree of reporting bias. In addition, we have been interested in CAS phenotype and laterality. The phenotype of symptoms likely mediated by cranial autonomic activation has emerged over time. One of these more recently identified symptoms is aural fullness [35–37], a sensation of the ear feeling full and uncomfortable, and sneezing [38, 39]. Whilst the majority of cranial vasculature innervation and therefore pain perception comes from V1 [24], we suggest based on previous work, that pain and CAS can be mediated via other divisions of the trigeminal nerve (in the absence of vascular dilatation) [30, 40].

In our experience in a specialist TAC clinic and in running a specialist Orofacial Pain clinic [41], patients with TAC’s and indeed migraine, do report pain outside of the V1 dermatome. We have witnessed the reporting of voice change and/or a sensation of throat swelling, which may be possible CAS, by patients affected by the primary headache disorders. These symptoms are not yet reported in the literature and may represent additional symptoms in the broadening CAS phenotype, perhaps associated with involvement of the V3 division of the trigeminal nerve.

In this study, we therefore sought to examine the prevalence and phenotype of CAS reported by migraineurs within our clinic, and to look at association with headache laterality and baseline headache frequency. We also aimed to evaluate the reporting of voice change and/or throat swelling as possible CAS within a separate cohort of those across migraine and TAC clinics, to evaluate how often these symptoms were reported, their co-occurrence with other CAS and association with pain site.

## Methods

We aimed to study the prevalence and phenotype of CAS among migraineurs within our clinical cohort, as well as the reporting of voice change and/or throat swelling as possible CAS among all primary headache disorders.

The study was conducted via review of the first outpatient clinic letter from first assessment within our service for selected patients, as this letter includes the details of a standardised detailed headache history taken by a trained headache physician, including age, headache diagnosis, baseline headache frequency and CAS phenotype. The general migraine cohort will be called cohort 1 and the smaller cohort of all primary headache disorders with voice change and/or throat swelling will be called cohort 2.

This study was considered a service evaluation of our history taking, and therefore, by UK guidance, did not require independent ethical approval. All data collected were anonymised before collation.

### Characteristics of participants

#### Cohort one

Migraineurs seen within our tertiary headache service between 2014 and 2018 were selected (*n* = 340). Clinical information regarding age, disease duration, sex, headache frequency, headache diagnosis, laterality of most severe headache and CAS phenotype was acquired from the first consultation clinic letter. For CAS laterality, the CAS were only deemed to be lateralised if all symptoms occurred unilaterally on the same side only. Headache was deemed unilateral (right or left), or bilateral, based on the laterality of most of the attacks and the most severe attacks decided by the patient.

#### Cohort two

First appointment clinic letters for patients seen within our service in any primary headache clinic between 2016 and May 2021, and letters containing the word ‘voice’ (*n* = 65), or ‘throat’ were selected (*n* = 151). Only those in which voice change and/or throat swelling were mentioned as CAS were analysed (*n* = 64). Information regarding patient age, headache diagnosis, pain site, preventive use and phenotype of reported CAS was collated.

### Statistical analysis

All data were collected anonymised, and an SPSS data sheet was populated. Descriptive statistical analyses were performed to assess the phenotype of CAS reported, and Pearson’s correlation coefficient was used to look for an association between headache frequency and number of CAS and Chi-square analysis to examine the relationship between headache laterality and CAS laterality (IBM SPSS v27). In addition, the co-occurrence of voice change and/or throat swelling as a possible CAS with other CAS in cohort two was assessed using Chi-square analysis.

## Results

### Cohort one

The mean age at evaluation was 43 years (range 14–94, SD 15), with a mean disease duration of 26 years (range 2–77, SD 14). The majority (84%) were female.

The most common diagnosis was chronic migraine (78%), and aura was present in 54%. The median number of headache days a month was 26 (range 1–30, IQR 15–75).

**Fig. 1:**
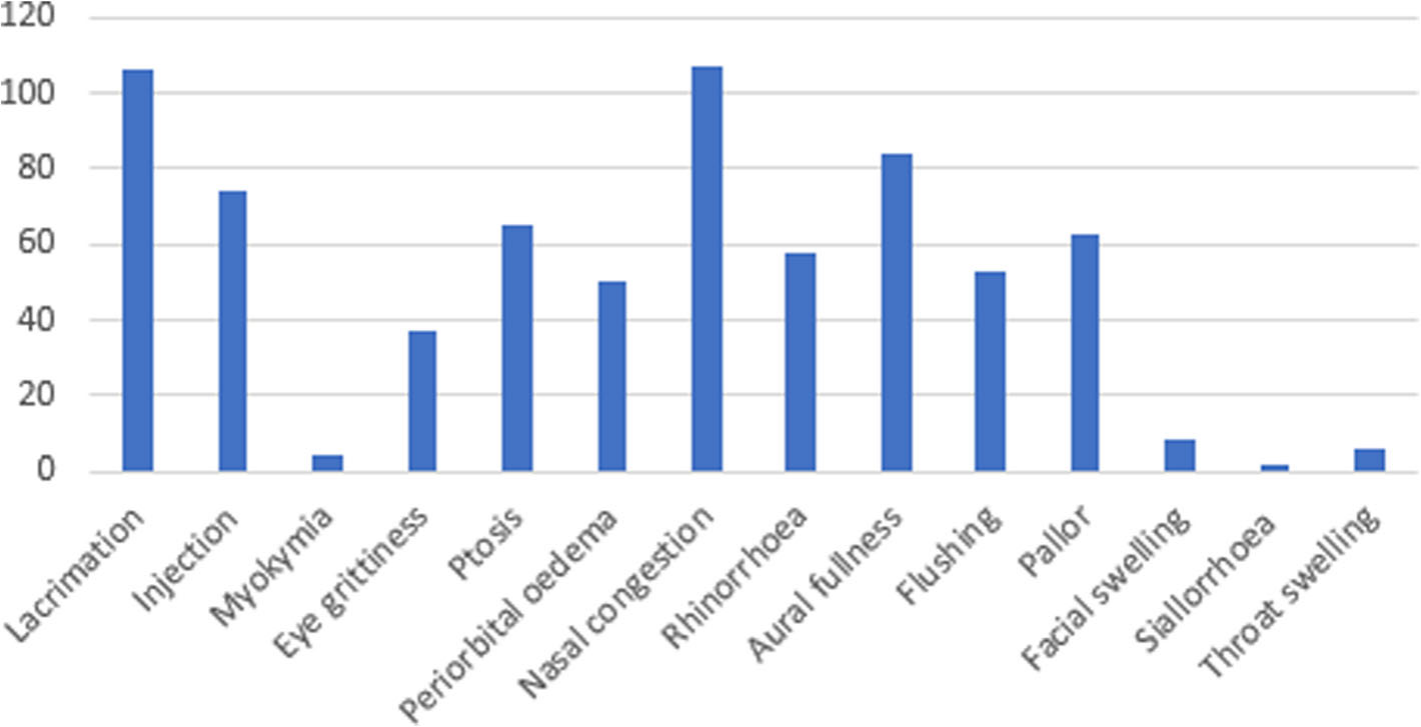
The phenotype of CAS reported in cohort 1. The y axis represents the number of patients reporting each symptom

At least one CAS was reported in 74%. A median of two CAS were reported (range 0–9, IQR 0–3), with the most common being nasal congestion (32%), lacrimation (31%) and aural fullness (25%). The complete phenotype of symptoms is shown in Fig. 1.

Most patients reported their most common headache was unilateral (80%) and with it, strictly unilateral CAS (64%). There was a positive association between laterality of severe headache and strict CAS laterality in the same individual (χ^2^_1 =_ 20.7, *P* < 0.001).

There was a weak positive correlation between headache frequency at baseline and the total number of CAS reported in any individual (*r* = 0.12, *P* = 0.047).

### Cohort two

Subjects included in the analysis (*n* = 64) were 72% female, age range 23–83 years (median 49, IQR 39–61).

**Fig. 2:**
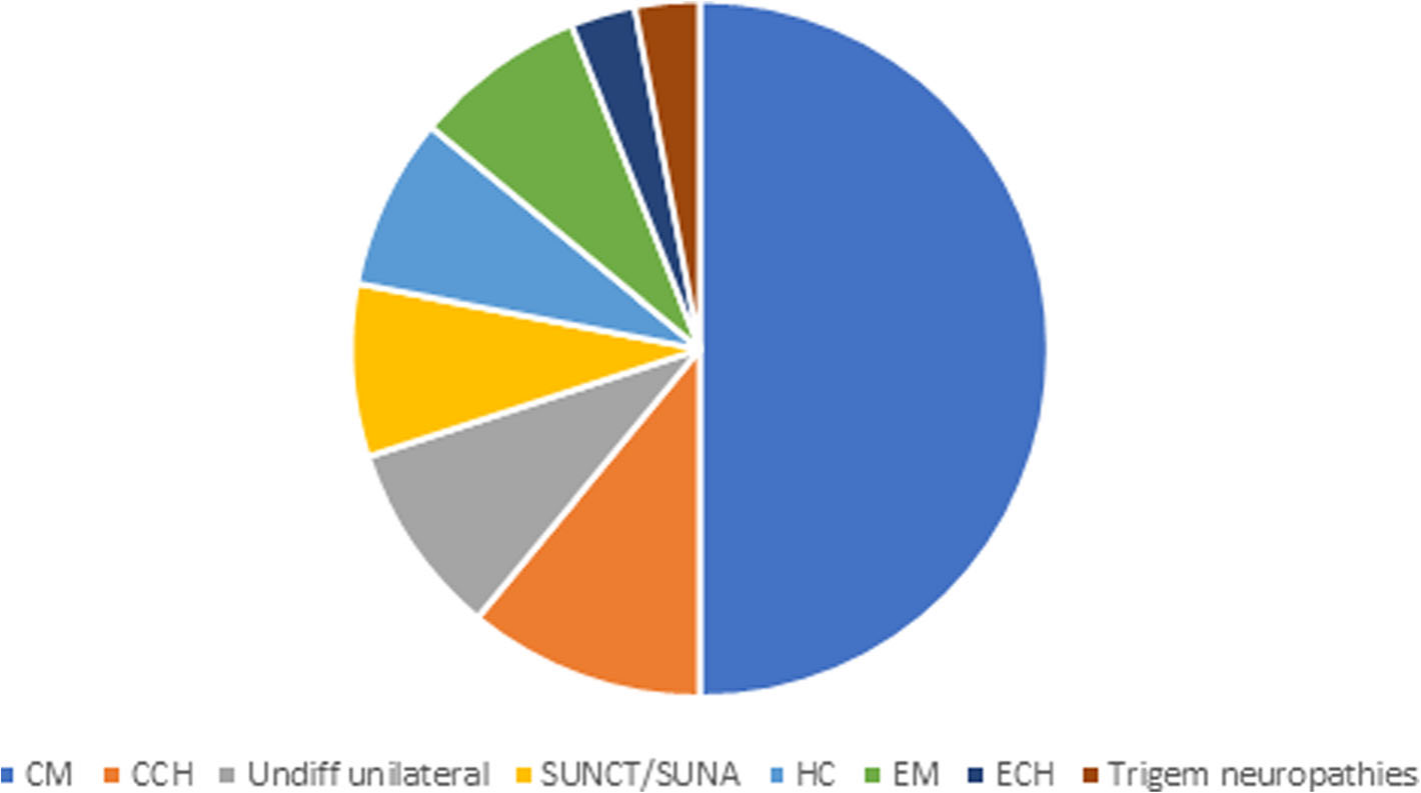
Summary of headache diagnoses in cohort 2. CM-chronic migraine, SUNCT- short-lasting unilateral neuralgiform headache with conjunctival injection and tearing, SUNA- short-lasting unilateral neuralgiform headache with cranial autonomic symptoms, ECH- episodic cluster headache, CCH- chronic cluster headache, HC- hemicrania continua, trigem neuropathies- trigeminal neuropathies, undiff unilateral- undifferentiated continuous lateralised headache, EM- episodic migraine

Headache diagnoses are summarised in Fig. 2.

**Fig. 3:**
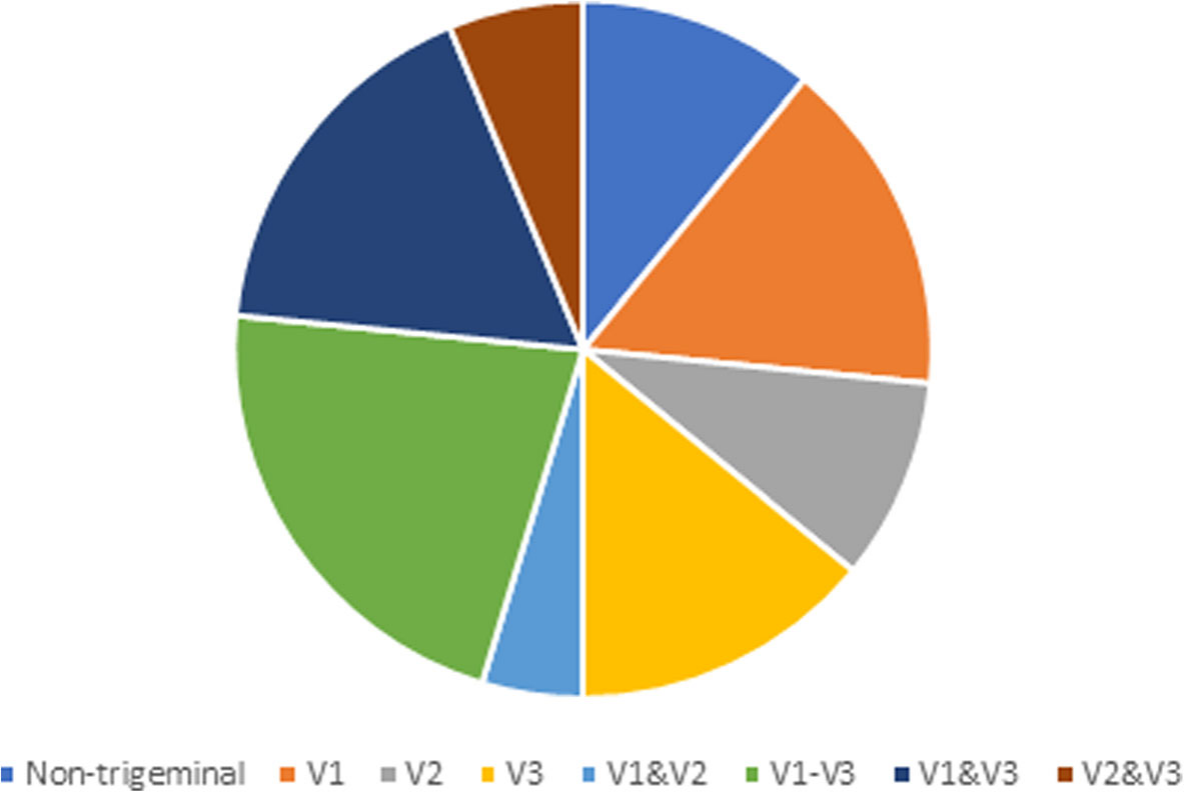
Summary of pain locations in cohort 2

The majority (89%) described pain in the distribution of the trigeminal nerve; 25% involving all three divisions, with 67% including V3. The pain sites (non-trigeminal distribution and the breakdown of trigeminal distribution pain) are shown in Fig. 3.

Less than half the patients (47%) were on headache preventive therapy; the preventive drugs taken are summarised in Table 1.

**Table 1:** Summary of preventive drugs taken by patients in cohort 2

Preventive drug(s)	Number of patients taking drug (***n***)
None	34
Fremanezumab	1
Carbamazepine and pregabalin	2
Melatonin and non-invasive vagal nerve stimulation	1
Pregabalin	2
Non-invasive vagal nerve stimulation	4
Botulinum toxin A	2
Candesartan	5
Gabapentin	1
Indomethacin	1
Amitriptyline	7
Verapamil	2
Carbamazepine	1
Topiramate	1

Throat swelling was reported by 54, voice change by 17, and both by 7.

**Fig. 4:**
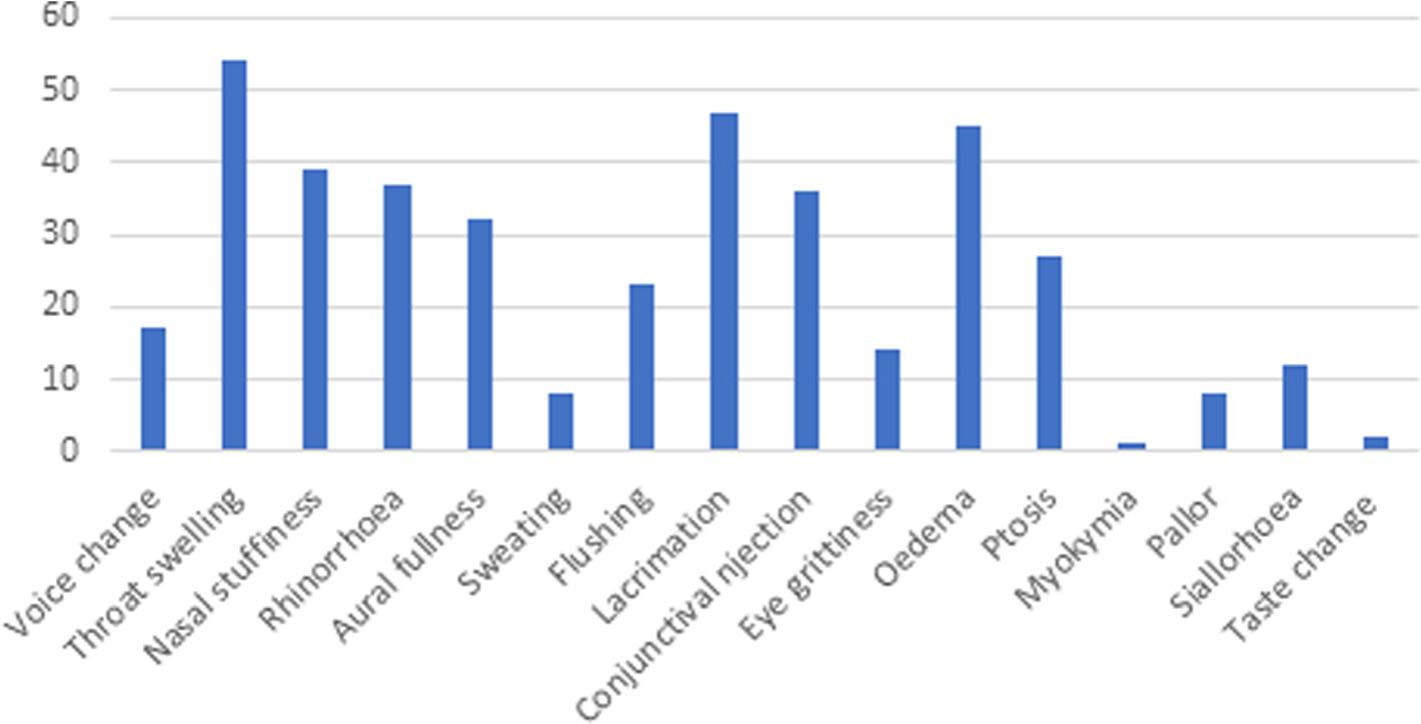
Summary of phenotype of CAS reported by cohort 2. The y axis represents the number of patients reporting each symptom

Between 1 and 11 CAS were reported (median 6, IQR 35–7); the most common were lacrimation (*n* = 47), facial swelling (*n* = 45) and rhinorrhoea (*n* = 37). The complete phenotype is shown in Fig. 4.

There was significant agreement between the co-reporting of throat swelling (χ^2^_1_ = 7.59, *P* = 0.013) and voice change (χ^2^_1_ = 6.49, *P* = 0.02) with aural fullness.

## Discussion

The data demonstrate a high frequency of reporting of CAS in migraine, with a varied CAS phenotype. There is a significant weak positive correlation between the number of CAS reported and baseline headache frequency, suggesting more symptoms associated with increasing headache burden. This has been previously reported [3, 4, 6, 9, 11].

There is also a statistically significant association between headache and CAS laterality in migraine, which has some nuanced difference to the existing literature [5, 7]: CAS do tend to lateralise to the side of headache in migraine when considering the most severe attacks, similarly to in the TAC’s. This may be the case because of the high proportion of chronic migraineurs and the high percentage of lateralised headache in the cohort studied. Some previous migraine studies also suggest more CAS in strictly unilateral and more severe headache [3, 4, 6, 9, 11], as well as an association with a higher degree of central sensitisation and allodynia [2]. There is a suggestion that CAS reporting increases with migraine chronification and that voice change and throat swelling are most commonly reported in chronic migraine, although this is the most represented group in this cohort studied in terms of patient numbers.

Voice change and/or throat swelling are not infrequently reported in our study, and may represent parasympathetically-mediated CAS, particularly given the majority of those reporting these symptoms have pain involving the third division of the trigeminal nerve. The co-association with aural fullness contributes to this idea that these symptoms may form part of a broadly somatotopic endophenotype, and this is an area that should be explored further.

The biological association between headache intensity and chronicity with CAS reporting is perhaps to be expected and suggests that the trigeminal autonomic pathway must be more engaged with increasing disease activity, as increasing TCC activation drives SSN activation. However, CAS have been reported in the absence of pain in both migraine [18] and cluster headache [20–23], so clearly pain is not a pre-requisite to their manifestation. Functional imaging work in migraine has suggest early involvement of the region of the dorsolateral pons prior to pain onset [42–44], and the superior salivatory nucleus (SSN) is located in this area. In addition, involvement of the hypothalamus, PAG and other areas has been suggested prior to pain onset in migraine [42], as have alterations in thalamocortical pathways [45], suggesting that the pathways involving the SSN and its descending modulation via higher brain structures, may be at play before pain onset in migraine and that activation of the SSN does not solely have to occur through nociception and TCC activation. It is therefore possible that top-down activation of the SSN and the autonomic pathway causes manifestation of CAS in the absence of pain in some individuals, and that activation of the pain pathway via the TCC feeds into this to worsen CAS in the presence of pain, and with increasing headache burden. Understanding these fundamental mechanisms of neurobiology of migraine and the TAC’s is vital to advancing therapeutics.

The identification of voice change, and perhaps the related sensation of throat swelling, as potential cranial autonomic symptoms further increase the heterogenous phenotype of CAS associated with the primary headache disorders. The suggested co-association of aural fullness, and the involvement of the V3 dermatome in the majority of cases of these symptoms, indicates that these symptoms may form part of a broadly somatotopic endophenotype; this is something that has not formally been reported before. Previous work has shown that painful capsaicin injection into the V1 area of the trigeminal nerve distribution causes pain in that area and accompanying dilatation of the selective intracranial portion of the internal carotid artery. In contrast, injection into V3 produces pain without accompanying vascular dilatation, suggesting that whilst there is an anatomical preference for V1 as the major contributor to cranial vasculature innervation, vessel dilatation is neither specific to primary headache disorders, nor necessary for pain perception. Trigeminal innervation of the neurovasculature is somatotopically organised and occurs in response to any pain [30]. Contributing to this theory is a study that showed persistence of cluster headaches with associated CAS in a patient who had undergone trigeminal root section on the side of pain [40], suggesting that absence of ipsilateral vessel dilatation mediated via trigeminal innervation is not necessary for pain perception or CAS mediation. Potentially highly somatotopically organised CAS may therefore be mediated by V2 and V3 also, without vascular dilatation being present. Further investigating the association between different CAS and pain locations systematically in the primary headache disorders (not just in suspected TAC’s) would help broaden this understanding and provide an anatomical and physiological correlate for many other under-reported possible CAS in headache practice. We have also witnessed the reporting of local areas of swelling outside of typical areas in the face (periorbital and cheek), including in areas of the scalp and neck, and parts of the gums, within our Headache and Orofacial Pain clinics, which may be similarly mediated somatotopically localised CAS.

This study has some limitations that are important to acknowledge. The two cohorts include different subjects, and the cohort for the possible new symptoms is small, as this was identified as a secondary objective when planning the evaluation. It is therefore difficult to draw definite conclusions from this cohort. We are keen to extend these observations in due course following adding these symptoms into our routine clinical questioning. In addition, there is a selection bias as all patients are from our tertiary clinics and therefore are more likely to have chronic and refractory headache disorders. Further work in different study populations with different headache burdens and medication use would help understand the true association of CAS with headache burden and the effect of migraine prevention on their reporting. In addition, our systematic history taking may lead to a degree of reporting bias of some CAS, but we feel that this form of questioning increases the likelihood of symptom capture and ensures the history elements are consistent from patient to patient. Further work in this area using population and primary care samples may give better ideas on CAS prevalence in migraine and help to evaluate further the broader CAS phenotype. We did not look to record the pain site and association with CAS phenotype in cohort one, but this is something we would aim to do going forwards, with the hope of contributing to the understanding of the anatomical mediation of different CAS depending on pain site. We hope to expand our analysis of patients by evaluating our Orofacial Pain clinic as these patients are more likely to have pain in the V2 and V3 areas of the face. We also did not examine the patient histories retrospectively to assess for change in CAS and their laterality for example prior to migraine chronification if applicable, and only the first interaction with the service was used for data capture as a snapshot for each included individual. Such data may be useful in case lateralisation changes or is less distinct when migraine evolves to chronic and the presence or absence of lateralised CAS in association with headache may have been more of a pertinent feature in the episodic form of the condition. Functional imaging of CAS, both in the absence of pain experimentally, and in the presence of pain, may offer important biological insights into the mediation of these symptoms.

## Conclusions

We suggest that CAS are common in migraine, similar in phenotype to the TAC’s and tend to lateralise with the pain in chronic lateralised migraine. Increasing CAS reporting is associated with increasing headache burden, although previous work in migraine and cluster headache suggest pain is not a prerequisite for their manifestation. The involvement of the SSN via top-down activation from higher brain structures such as hypothalamus, at least in some sufferers, is likely to occur, suggesting TCC activation is not the only means of stimulating the cranial autonomic pathway. We also propose that two further possibly associated symptoms: voice change and a sensation of throat swelling, may be parasympathetically-mediated CAS, which are under-reported in clinical practice, and may be co-associated with aural fullness. This co-association, and the symptoms being mostly present in those reporting pain involving the V3 dermatome, suggests that these symptoms and perhaps other CAS too, contribute to a broadly somatotopic endophenotype, mediated by all three divisions of the trigeminal nerve in the absence of vascular dilatation. This would suggest that further systematic questioning about CAS phenotype and pain site in all the primary headache disorders may yield interesting insights into how these symptoms are mediated, and therefore into the fundamental neurobiology of these disorders.

## Abbreviations

CAS: Cranial autonomic symptoms
CCH: Chronic cluster headache
CM: Chronic migraine
ECH: Episodic cluster headache
EM: Episodic migraine
HC: Hemicrania continua
LC: Locus coeruleus
PAG: Periaqueductal grey
SSN: Superior salivatory nucleus
SUNCT: Short-lasting unilateral neuralgiform headache with conjunctival tearing and lacrimation
SUNA: Short-lasting unilateral neuralgiform headache with autonomic features
TAC: Trigeminal autonomic cephalalgia
TCC: Trigeminocervical complex
TG: Trigeminal ganglion
VIP: Vasoactive intestinal peptide
